# Editorial: Nutritional physiology and gut microbiome

**DOI:** 10.3389/fmicb.2023.1227522

**Published:** 2023-06-13

**Authors:** Konstantinos Papadimitriou, Tingtao Chen, Peng Huang, Jia Yin

**Affiliations:** ^1^Laboratory of Food Quality Control and Hygiene, Department of Food Science and Human Nutrition, Agricultural University of Athens, Athens, Greece; ^2^Institute of Translational Medicine, Nanchang University, Nanchang, China; ^3^College of Animal Science and Technology, Hunan Agricultural University, Changsha, China; ^4^Hunan Key Laboratory of Traditional Chinese Veterinary Medicine, Hunan Agricultural University, Changsha, China; ^5^Hunan Provincial Key Laboratory of Animal Intestinal Function and Regulation, College of Life Sciences, Hunan Normal University, Changsha, China; ^6^Hunan International Joint Laboratory of Animal Intestinal Ecology and Health, College of Life Science, Hunan Normal University, Changsha, China

**Keywords:** gut microbiome, animal nutrition, host metabolism, multi-omics analysis, host health

The gut microbiota is the largest symbiotic ecosystem in the host and has been demonstrated to play an important role in maintaining intestinal homeostasis. The symbiotic relationship between the microbiota and the host is mutually beneficial. The host provides important habitat and nutrients for the microbiome. The gut microbiota supports the development of the metabolic system and the intestinal immune system's maturation. Intestinal microbes ingest dietary components such as carbohydrates, proteins, and lipids, and the metabolites are reported to directly or indirectly affect human health. Therefore, there is an inseparable relationship between the gut microbiota and the nutrition of the host.

Gut microbiota not only participates in the digestion, absorption, and synthesis of some nutrients but also regulates host metabolism. A detailed understanding of this relationship between gut microbiota and animal nutrition physiology is necessary to rationalize dietary interventions targeted at the gut microbiota in the future. The emergence of omics methods and research on the animal microbiome has completely changed our understanding of gut microbiota and nutritional physiology. This Research Topic (RT) focuses on all aspects of the research on digestive tract microorganisms and nutritional physiology.

Various research articles submitted on this RT focus on the impact of specific dietary interventions on the microbiome and the physiology of certain farming animals, including pigs and piglets, lambs and goats, ducks, chickens, bullfrogs, and shrimps. Yang, Wang, et al. explored the impact of different dietary ratios of amylose and amylopectin on the gut health and microbiome of weaned pigs during feed transitions or after exposure to the lipopolysaccharide toxin of *Escherichia coli*. Wang M. et al. investigated the effects of an herbal extract mixture on the gut microbiota and intestinal antioxidant capacity of weaning piglets. Zhou H. et al. researched the effects of administering all-trans retinoic acid (ATRA) to pregnant sows on the gut bacterial community of neonatal piglets with different genetic backgrounds. Zheng et al. looked into the mechanisms underlying the effects of beta-hydroxy-beta-methylbutyrate (HMB) on lipid metabolism in Bama Xiang mini-pigs. Li et al. compared the effects of compound enzymes and antibiotics on growth performance, nutrient digestibility, blood biochemical index, and intestinal health in weaned pigs. All these studies demonstrated that the dietary interventions could lead to alterations in the gut microbiome of pigs and piglets, suggesting improved gut health and physiological functions like resistance to bacterial toxins, enhanced anti-oxidative capacity, growth performance, etc.

Furthermore, Wang Q. et al. assessed the impact of different dietary energy levels in male Hu lambs during the fattening period, and Yang, Zhang, et al. assessed the effects of dietary supplementation with mannan oligosaccharide (MOS) on the passive transfer of immunoglobulin G (IgG), anti-oxidative capacity, immunity, and intestinal microbiota in neonatal goats. The first study suggested that the medium rather than the high dietary energy levels could be more appropriate during the lamb fattening period based on the changes observed in rumen fermentation, gastrointestinal tract histology, and microbiome diversity. The second study concluded that MOS could improve all parameters tested and thus its addition as a feed supplement for neonatal goats is suggested.

An additional three studies focused on dietary improvements in poultry. In ducks, Liu et al. and Peng et al. explored the effects on gut microbiota, growth performance, and intestinal morphology among other physiological parameters of dietary ferulic acid or *Bacillus* and non-starch polysaccharase, respectively. He et al. studied the effects of incorporating black soldier fly larvae meal (BSFLM) into the diets of Xuefeng Black-Bone chickens on their gut microbiota and intestinal morphology. The studies mentioned above indicated that the dietary interventions tested had positive effects on the gut health and productivity of poultry.

The final two studies on farming animals were on organisms related to aquaculture. Wang Z. et al. analyzed the effects of supplementing autochthonous gut bacteria in plant-based diets on the growth, nutrient digestibility, and gut health of bullfrogs (*Lithobates catesbeianus*). Wang W. et al. looked at the effects of dietary supplementation with *Phaffia rhodozyma* astaxanthin on growth performance, carotenoid analysis, biochemical and immune-physiological parameters, gut microbiota, and disease resistance in the giant tiger prawn *Penaeus monodon*. Again, a positive influence was recorded for each of the interventions in the two organisms which could be related to the gut microbiome, gut physiology, and growth performance.

The next five studies were performed in mice or rats as the model organisms. Wen et al. investigated the effects of heat stress on the gut microbiota and metabolomic profiles in mice. Heat stress was found to have a negative effect on the gut microbiome composition and caused metabolic alterations which could be related to inflammation and oxidative stress. Zhuang et al. studied the effects of resveratrol on growth performance, intestinal morphology, gut microbiota composition, and metabolism in mice. Zhai et al. unraveled the mechanisms underlying the effects of *Eucommia ulmoides* leaf extract (EULE) on colonic epithelium integrity in rats. Zhou D. et al. tested the application of phytic acid-degrading bacteria on mineral element content in mice. Wan et al. checked the effects of caffeic acid supplementation on colonic inflammation, oxidative stress, and gut microbiota in mice. Resveratrol had a positive impact on all physiological and morphological parameters tested. EULE supplementation led to improvements in colonic epithelium integrity, accompanied by a reduction in inflammation and oxidative stress. EULE action was found to be mediated through the gut microbiota-bile acids-TGR5 axis. Degradation of the anti-nutrient phytic acid, which binds to minerals and inhibits their absorption in the gut, led to their increased absorption and utilization. This effect was accompanied by positive alterations in the composition of the gut microbiome. Moreover, caffeic acid supplementation led to reductions in colonic inflammation and oxidative stress along with positive changes in the gut microbiota composition. The antioxidant and anti-inflammatory effects of caffeic acid were associated with changes in the level of gene expression.

Finally, three review articles were published in the RT. The first concentrated on the potential use of *Lonicera japonica* extracts (LJE) in animal production, with a focus on their effects on intestinal health. In this review, Tang et al. discussed the mechanisms underlying the positive effects of LJE on the gut microbiota, gut barrier function, and immune function. Overall, the review suggested that LJE has potential applications in animal production for promoting gut health, enhancing animal performance, and reducing the need for antibiotics. In the second review, Yang Y. et al. presented recent research on the relationship between cow's milk protein allergy (CMPA) and the gut microbiota in infants. The authors suggested a relation between CMPA and gut dysbiosis, as well as the potential use of probiotics, prebiotics, and synbiotics in the prevention and management of CMPA. In the third and final review, Hu et al. discussed the potential applications and functions of prophage activation in the intestine. The study highlighted that prophage activation could play a role in the modulation of gut microbiota composition and function, in the treatment of disease (e.g., inflammatory bowel disease) and infection (e.g., *Clostridioides difficile*), as well as in engineering the microbiome (e.g., production of therapeutic compounds).

The studies featured in this RT encompass significant contributions from diverse authors, highlighting the multifaceted aspects between nutritional physiology and the gut microbiome in animal and human health.

## Author contributions

All authors listed have made a substantial, direct, and intellectual contribution to the work and approved it for publication.

